# Supplementary figures and images for: The Use of Stable Isotope Ratio Analysis to Trace European Sea Bass (D. labrax) Originating from Different Farming Systems
Source: Animals (Basel). 2020 Nov 5;10(11):2042. doi: 10.3390/ani10112042 (PMC7694367; doi:10.3390/ani10112042)

(a)

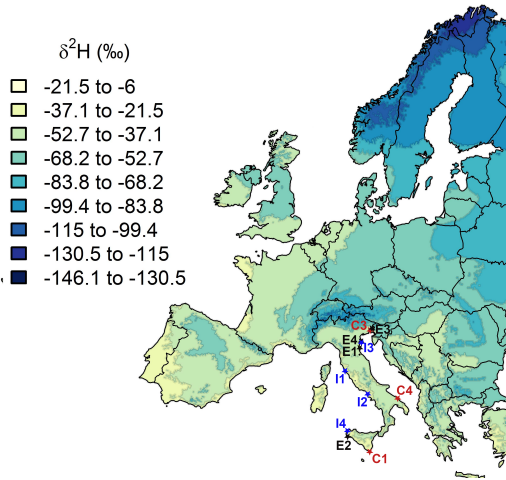

(b)

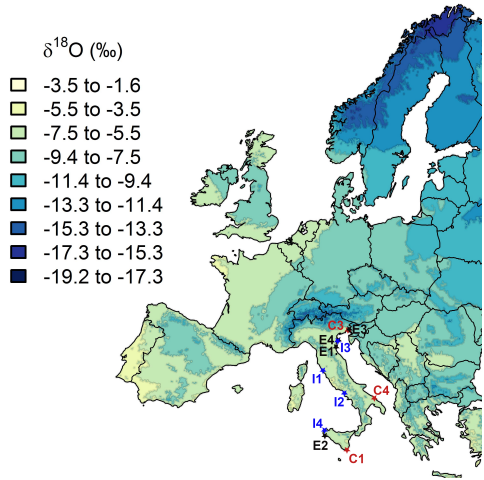

Supplement: Supplementary file 1 [file animals-10-02042-s001.pdf]
